# Identification and antimicrobial susceptibility profile of *Escherichia coli* isolated from backyard chicken in and around ambo, Central Ethiopia

**DOI:** 10.1186/s12917-019-1830-z

**Published:** 2019-03-11

**Authors:** Edilu Jorga Sarba, Kebede Abdisa Kelbesa, Morka Dandecha Bayu, Endrias Zewdu Gebremedhin, Bizunesh Mideksa Borena, Ayichew Teshale

**Affiliations:** 1 Department of Veterinary Sciences, Ambo University, P O Box 19, Ambo, Ethiopia; 2 Department of Veterinary Laboratory Technology, Ambo University, P O Box 19, Ambo, Ethiopia; 3 School of Veterinary Medicine, Wolaita Sodo University, P O Box 138, Sodo, Ethiopia

**Keywords:** Ambo, Antimicrobial resistance, Chicken, *E. coli*, Visceral organ

## Abstract

**Background:**

*Escherichia coli* is bacteria that exist as commensal in the intestine of animals and humans, but pathogenic strains cause disease in chickens. The development of antimicrobial resistance in *E. coli* is one of major concern worldwide. A cross-sectional study was conducted from November, 2015 to April, 2016 in and around Ambo town on backyard chicken with the objectives of isolating *E. coli* from selected visceral organs, assessment of potential risk factor and determination of antimicrobial resistance pattern of the isolates.

**Results:**

The overall isolation rate of *E. coli* was 11.5% (80/694) [95% CI: 9.64–14.61] and 32.5% (62/191) [95% CI: 25.39–39.09] at organ and chicken level, respectively. *E. coli* isolation rate was 15.2% (29/191), 13.6% (27/191), 6.3% (12/191) and 10.7% (13/121) from spleen, liver, kidney and ovary samples, respectively. The multivariable logistic regression analysis revealed higher probability of *E. coli* isolation from adult (adjusted Odds ratio [aOR] =2.5, *P* = 0.013) than younger chickens, from clinically sick chickens (aOR = 3.0, *P* = 0.003) than apparently healthy. *E. coli* isolates were 100% susceptible to ciprofloxacin, norfloxacin and sulfamethoxazole-trimethoprim followed by 89–63.4% susceptibility to gentamicin, streptomycin, ceftazidime, nalidxic acid, nitrofurantoin, kanamycin, amikacin and chloramphenicol. Whereas, 100% resistance was observed against cloxacilin, cefotaxime and amoxicillin, whereas 92.7 and 46.3% were resistant to cefuroxime, and tetracycline, respectively. Multidrug resistant (MDR) was observed in 78.1% (64/82) of the isolates which exhibited 5 different MDR patterns to 7 antimicrobial classes.

**Conclusions:**

Higher isolation rate of *E. coli* was observed from visceral organs of chickens. Age and health status were predictors of *E. coli* isolation. Remarkable numbers of the isolates are resistant to different antimicrobials and multidrug resistant *E coli* isolates are widespread in the area.

**Electronic supplementary material:**

The online version of this article (10.1186/s12917-019-1830-z) contains supplementary material, which is available to authorized users.

## Background

Ethiopia owns an estimated chicken population of 51.35 million with native chicken breeds representing 96.6%, and the remaining 0.55 and 2.8% are hybrid chickens and exotic breeds mainly kept in urban and peri-urban areas, respectively [1]. The backyard poultry represents an important part of the national economy and provides about 98·5% and 99·2% of national egg and poultry meat production, respectively [2]. Inadequate knowledge about poultry production, limited feed resources, low productivity of indigenous chicken breeds, high prevalence of diseases and predation are among the constraints of backyard poultry production in Ethiopia [3]. Colibacillosis, salmonellosis, mycoplasmosis and fowl cholera are among the major bacterial diseases which threaten the poultry industry all over the world, including Ethiopia.

*Escherichia coli* (*E. coli*) is considered as a member of the normal microflora of all warm-blooded animals including poultry [4]. However, in the debilitated or in immune suppressed hosts, or when gastro-intestinal barriers are violated, even normal “non-pathogenic” strain of *E. coli* can cause infection to poultry, humans and animals. Moreover, there are certain *E. coli* strains designated as avian pathogenic *E. coli*, spread into various internal organs and cause colibacillosis characterized by systemic fatal disease [5]. Diseases associated with *E. coli* in poultry are manifested by yolk sac infection, omphalitis, respiratory tract infection, septicaemia, polyserositis, enteritis, cellulitis and salpingitis [6]. Pathogenic *E. coli* strains are those possessing one or more virulence factors and the most common isolates in poultry belong to O78, O1, and O2, and to some extent O15 and O55 sero-groups. In domestic poultry, avian colibacillosis is frequently associated with *E. coli* strains of serotypes O78:K80, O1:K1 and O2:K1 [7].

On the other hand, antimicrobial resistance associated with inappropriate use of antimicrobial drugs in humans and animals has been the major factor for the emergence and spread of drug-resistance traits among pathogenic and commensal bacteria. The development of multi-drug resistance in *E. coli* is one of major concern worldwide [8]. In Ethiopia veterinary drugs are regulated by Veterinary Drug and Animal Feed Administration and control Authority (VDFACA) following the proclamation No.728/ 2011. The national drug list serves as a guide for registration, procurement, distribution and prescription of veterinary drugs in the country. However, the veterinary drug regulation and guidelines are not so well developed and not enforced to the standard so as to practice responsible and prudent use of antimicrobials in veterinary medicine. Due to this not only indiscriminate use of antimicrobials is common but also sale and distribution of counterfeit antimicrobials and the sale of antimicrobials on the informal market and involvement of untrained persons in the profession are big challenges [9]. Concerning the classes of antimicrobials prescribed for veterinary use in Ethiopia, reports from Adama and Bishoftu areas of central Ethiopia shows that oxytetracyclines of various formulations are the most commonly prescribed antimicrobial followed by penicillin-streptomycin fixed combination, sulpha drugs (sulphadimidine and sulphametoxazole-trimethoprim fixed combination), procaine penicillin, penicillin + cloxacilin, chloramphenicol, neomycin sulphate (intra-mammary infusion) and gentamicin [10, 11]. According to the above authors, almost all (100%) cases in district veterinary clinics receive antimicrobial therapy after they had been tentatively diagnosed. These are indicative of irrational use of antimicrobials which are pre-requisites to an increase in the resistance of microorganisms to commonly used drugs.

Although there are few reports on the prevalence of chicken diseases in Ethiopia, studies regarding the antimicrobial susceptibility profile of *E. coli* isolated from chicken of backyard origin is scarce in Ethiopia. Thus, there is a need to study the rate of *E. coli* isolation in different chicken organs, its possible association with the risk factors and antimicrobial resistance pattern for better understanding of the situation in the study area. Therefore, the objectives of this study were to isolate *E. coli* from apparently healthy and clinically sick chickens, to identify the risk factors associated with *E. coli* infection and determine the antimicrobial resistance pattern of isolates.

## Materials and methods

### Description of study area

Chickens for this study were purchased from local markets of Ambo, Holeta, Guder, Ijaji and Dire Inchini districts of the West Shewa Zone, Oromia Regional State of Ethiopia from January 2016 to April 2017. Ambo is the administrative centre of the Zone, which is located at 114 Kms West of Addis Ababa. The altitude of Ambo is midland. Holeta and Dire Inchini are located 70 Kms East and 40 Kms southwest of Ambo, respectively and both are in highland altitude range. Guder and Ijaji are located 15 Km and 80 Km West of Ambo and both have tropical climate. The chicken population of each district is approximately between 350, 000 to 500,000 [12].

### Study animals and their management

The study animals were backyard chickens that are kept under extensive management system, where chicken scavenge their feed the whole day with a limited supplement and often share same house with humans or other livestock. In this system, chickens are not vaccinated and veterinary service is not well developed. In this study, apparently healthy and clinically sick and culled chickens were purchased from local markets. Chickens of both sexes and local and hybrid breeds managed under backyard system were included. Chickens were categorized as young (≤6 months) and adult (> 6 months) based on their age [13] and as clinically sick and apparently healthy based on presence or absence of clinical signs of diseases.

Following purchase, chickens were transported in a cage with adequate space and ventilation without exposing to extreme weather condition. Sick and dead chickens were transported separately and slaughtered immediately. Following arrival, chickens were kept in Ambo University in a house with adequate living space and ventilation for a maximum of one day before slaughter. Feed (wheat and cracked corn) and clean water was ad libitum.

### Study design, sampling technique and sample collection

A cross-sectional study design was used and a total of 191 chickens were selected consisting of apparently healthy (*n* = 95) and clinically sick or dead (*n* = 96). Apparently healthy chickens were selected randomly from local markets, while clinically sick chickens (chickens with diarrhoea, loose appetite, depression etc...) were purchased purposively from traders and farmers. All chickens were physically examined for their health status and subjected to post-mortem examination. Cervical dislocation was used to euthanize chickens in a humane manner. The carcasses were promptly necropsied according to the standard procedures described by Lowenstine [14]. During necropsy, a total of 694 visceral organ samples of liver (*n* = 191), spleen (n = 191), kidney (n = 191) and ovaries (*n* = 121) were sampled. About 25 g of each organ sample was collected from the internal portion aseptically in sterile plastic bag (Falconpack, UAE). Samples were kept at + 4 °C for a maximum of 24 until culturing. Bacteriological work was done in the Veterinary Microbiology Laboratory of Ambo University.

### Isolation and identification of *E. coli*

Isolation of *E. coli* was performed using standard bacteriological methods [15]. Organ samples were crushed by gentle maceration, mixed separately with buffered peptone water (BPW) and incubated at 37 °C for overnight. A loopful of the culture suspension was streaked onto MacConkey agar (HiMedia, Pvt. Ltd., India) and incubated for 24 h at 37 °C aerobically. The next day those pink coloured presumptive *E. coli* colonies were sub-cultured onto nutrient agar to get a pure colony, followed by sub-culture on Eosin Methylene Blue (EMB) agar (HiMedia, Pvt. Ltd., India). Colonies with metallic green sheen on EMB were later characterized microscopically using Gram’s stain. Putative *E. coli* colonies were then transferred onto nutrient agar for further identification using biochemical tests. Triple sugar iron (TSI) agar (HiMedia, Pvt. Ltd., India) was used for further characterization. Observation of yellow slant, yellow butt, presence of gas bubbles, and absence of black precipitate in the butt was considered as potentially *E. coli* isolate. Then the isolates were subjected to different biochemical tests such as indole production, methyl-red, Voges- Proskauer, citrate utilization (IMViC) and motility tests as per Quinn et al. [15]. *E. coli* ATCC 35218 (obtained from Ethiopian public health institute) was used as a reference organism.

### Antimicrobial susceptibility test

The antimicrobial susceptibility testing of *E. coli* isolates was conducted using Kirby- Bauer disc diffusion method on Mueller-Hinton agar (HiMedia, Pvt. Ltd., India) according to the guidelines of the Clinical and Laboratory Standards Institute [16]. All *E. coli* isolates were evaluated for antimicrobial susceptibility using 16 antimicrobials (9 antimicrobial classes) commonly used in veterinary and public health sectors in Ethiopia. Accordingly, a McFarland 0.5 standardized suspension of the bacteria in tryptone soya broth (HiMedia, Pvt. Ltd., India) was prepared and incubated for 6–8 h and using sterile cotton swab streaked over the entire surface of Mueller-Hinton agar. A ring of discs containing known concentrations of each antimicrobial drug was then placed onto the inoculum surface using disc dispenser, gently pressed with the point of the forceps for ensuring complete contact with the agar surface and incubated at 37 °C aerobically for 16–18 h. Clear zones of bacterial growth inhibition were measured in mm using a measuring calliper. The antimicrobials and their concentrations used for the susceptibility testing were streptomycin (10 μg), kanamycin (30 μg), gentamicin (30μg), amikacin (30 μg), amoxicillin (20 μg), cloxcillin (5 μg), cefuroxime, ceftazidime (30 μg), cefotaxime (30 μg), chloramphenicol (30μg), ciprofloxacin (5μg), nalidixic acid (30μg), nitrofurantoin (10 μg), tetracycline (30 μg), sulfamethoxazole-trimethoprim (1.25/23.75μg), and norfloxacin (10 μg) (Oxoid Ltd., Cambridge, UK). *E. coli* ATCC 35218 which is susceptible to all the drugs was used as a quality control. Finally, the findings were recorded as susceptible, intermediate, and resistant according to Clinical and Laboratory Standards Institute [16] break points.

### Data management and analysis

Data collected from questionnaire survey and laboratory study were entered in to Microsoft Excel () Spread sheet and analysed using STATA version 11.0 for windows (Stata corp. College Station, TX, USA). Descriptive statistics was utilized to summarize the data using percentages. The prevalence of *E. coli* with respect to district, sex, age, and season, health status, and diarrhoea were computed by dividing the number of positive chickens by the number of chickens examined and for organ level prevalence the number of positive organs was divided to the total number of organs examined. The association of potential risk factors with *E. coli* prevalence was analysed using logistic regression. Stratification method was used for those variables showing significant association to see any difference between the crude and adjusted results. Then, after further checking for collinearity, variables with *P*-value less than 0.25 during univariable analysis were further analysed using a multivariable logistic regression model. Odds ratio was used to see degree of association and confidence level was held at 95% and significance was at *P* < 0.05. The percentages of antimicrobial resistance of each pattern (Susceptible, Intermediate and Resistance) were calculated.

## Results

### Prevalence of *E. coli* at animal level

Out of the 191 chickens examined *E. coli* was isolated from 62 chickens (32.5%) [95% confidence interval (CI): 25.39–39.05%].

### Distribution pattern of *E. coli* isolates in different visceral organ

From the 694 organ samples examined, *E. coli* was isolated from 80 (11.5%) organs [95% CI: 9.14–14.1%]. There was a variation in the isolation rate of *E. coli* between organs with the highest rate observed in spleen 29/191(15.2%), followed by liver 26/191(13.5%), ovary 13/121(10.7%) and the lowest in kidneys 12/191(6.3%) [*P* > 0.05].

### Association of risk factors with the isolation rate of *E. coli*

Breed, sex, age, districts, season of the year, presence of diarrhoea, and health status were computed for any association with prevalence of *E. coli*. Univariable logistic regression analysis showed that age, health status and diarrhoea were significantly associated (*P* < 0.05) with isolation rate of *E. coli*. Accordingly, rate of *E. coli* isolation was significantly higher in adult (OR = 1.96, *P* = 0.044) than young chickens. Clinically sick chickens (OR = 2.44, *P* = 0.005) and those with diarrhoea (OR = 2.12, *P* = 0.017) are more likely to be *E. coli* positive as compared to apparently healthy and non-diarrheic chicken, respectively. Breed, sex, district and season didn’t show significant association (*P* > 0.05) and were excluded from the final model due to high univariable *P*-value (Table 1). All the variables were checked for collinearity, except for health status and diarrheic status (r = 0.81) and the rest were not colinear (r < 0.3). After checking for confounding using stratification method, since there was no difference between the crude and adjusted results, health status and age were selected to enter into multivariable logistic regression model. It was also observed that 20.4% of *E. coli* isolated were from single organ (*n* = 39), 10.5% from two organs (*n* = 20) and 1% from three organs (n = 2) per chicken. Although not statistically significant, all the chickens from which *E. coli* was isolated from three organs were diarrheic and sick. Similarly, majority of the chickens 80% (16/20) from which *E. coli* was isolated from two organs were clinically sick (Data not shown).

**Table 1:** Logistic regression analyses of the risk factors for isolation of *E. coli* in chicken

Risk factor	Category	No. Examined	No. Positive (%)	Univariable		Multivariable	
				OR (95% CI)	P. value	OR (95% CI)	P. value
Breed	Exotic	28	8 (28.6)	1.0		–	–
	Local	163	54 (33.1)	1.23 (0.51–2.99)	0.0635		
Age	Young	72	17 (23.6)	1.0		1.0	
	Adult	119	45 (37.8)	1.96 (1.02–3.79)	0.044	2.5 (1.22–5.11)	0.012
Sex	Male	70	22 (31.4)				
	Female	121	40 (33.1)	0.92 (0.49–1.74)	0.817	–	–
Season	Dry	83	23 (27.7)	1.0			
	Wet	108	39 (36.1)	0.68 (0.36–1.26)	0.220	0.74 (0.27–1.97)	0.550
District	Guder & Ijaji	55	14 (25.5)	1.0		1.0	
	Dire inchicni	40	11 (27.5)	1.11 (0.44–2.79)	0.823	2.1 (0.67–6.49)	0.200
	Ambo	64	23 (35.9)	1.19 (0.74–3.63)	0.220	0.57 (0.43–3.27)	0.724
	Holeta	32	14 (43.75)	2.28 (0.90–5.75)	0.081	2.22 (0.83–5.90)	0.110
Health status	Healthy	95	22 (22.1)	1.0	–		
	Sick	96	40 (42.7)	2.44 (1.31–4.57)	0.005	3.28 (1.54–6.96)	0.002
Diarrhoea	Absent	113	29 (25.7)	1.0			
		78	33 (41.3)	2.12 (1.15–3.93)	0.017		

### Antimicrobial susceptibility testing of *E. coli* isolates

The results of antimicrobial susceptibility test showed that there was variation in susceptibility of *E. coli* isolates to the drugs used. *E. coli* isolates revealed high susceptibility (100%) to ciprofloxacin, sulfamethoxazole-trimethoprim and norfloxacin followed by gentamicin (89%), streptomycin (85%), ceftazidime (84.6%), nalidxic acid (83%), nitrofurantoin (76.8%), kanamycin (75%), amikacin (69.6%) and chloramphenicol (63.4%). *E. coli* were resistant to 12 of the 17 antimicrobials tested. Higher resistance (100%) was observed to cloxacillin, cefuroxime and amoxicillin followed by cefotaxime (92.7%), tetracycline (46.3%), nitrofurantoin (23.2) and chloramphenicol (17.1%) [Table 2].

**Table 2:** Antimicrobial resistance pattern of *E. coli* isolates from chicken visceral organs

Antimicrobial class	Antimicrobial discs and concentration	*E. coli* isolates (*n* = 82)		
		No susceptible (%)	No Intermediate (%)	No Resistant (%)
Aminoglycosides	Streptomycin (10 μg)	70 (85)	12 (15)	0 (0)
	Kanamycin (30 μg)	62 (75)	12 (15)	8 (10)
	Gentamicin (30 μg)	73 (89)	9 (11)	0 (0)
	Amikacin (30 μg)	57 (69.6)	19 (23.1)	6 (7.3)
β-lactams	Amoxicillin (20 μg)	0 (0)	0 (0)	82 (100)
	Cloxcillin (5 μg)	0 (0)	0 (0)	82 (100)
Cephems	Cefuroxime (30 μg)	0 (0)	0 (0)	82 (100)
	Ceftazidime (30 μg)	69 (84.6)	0 (0)	13 (15.4)
	Cefotaxime (30 μg)	0 (0)	6 (7.3)	76 (92.7)
Phenicols	Chloramphenicol (30 μg)	52 (63.4)	16 (19.5)	14 (17.1)
Quinolones	Ciprofloxacin (5 μg)	82 (100)	0 (0)	0 (0)
	Nalidxic acid (30 μg)	68 (83)	8 (9.7)	6 (7.3)
Nitrofurans	Nitrofurantoin (10 μg)	63 (76.8)	1 (1.2)	18 (23.2)
Tetracyclines	Tetracycline (30 μg)	44 (53.7)	0 (0)	38 (46.3)
Folate pathway inhibitors	Sulfamethoxazole-trimethoprim (1.25/23.75 μg)	82 (100)	0 (0)	0 (0)
Fluoroquinolones	Norfloxacin (10 μg)	82 (100)	0 (0)	0 (0)

Among the resistant *E. coli*, 78.1% (64/82) were multidrug resistant (MDR) and exhibited 5 different MDR patterns to 7 antimicrobial classes (Table 3). MDR *E. coli* were resistant to as few as two and as many as 7 antimicrobials classes. All the 82 *E. coli* isolates were resistant to one of the β-lactams and Cephems, while 10 of the *E. coli* were resistant to 7 antimicrobial classes (β-lactams, Cephems, Macrolides, Tetracycline, Phenicols, Nitrofurantoin, Aminoglycosides and Quinolones).

**Table 3:** Multidrug resistance patterns in *E. coli* isolated from chicken visceral organs

Number	Antimicrobial resistance pattern	No. of resistant isolates (%)
Two	AMX CRX	34 (41.5)
Four	AMX CRX CHL	10 (12.2)
Four	AMX CRX TET	20 (24.4)
Five	AMX CRX TET NIT	8 (9.8)
Seven	AMX CRX TET NIT KN NLD	6 (7.3)
Seven	AMX CRX TET NIT KN CHL	4 (4.9)

AMX-Amoxicillin (β-lactams), CRX-Cefuroxime (Cephems), TET-Tetracycline, CHL- Chloramphinicol (Phenicols), NIT-Nitrofurantoin, KN-Kanaycin (Aminoglcosides), NLD- Nalidxic acid (Quinolones)

## Discussion

In this study *E. coli* was isolated from spleen (15.2%), liver (13.6%), ovary (10.7%) and kidney (6.3%). This finding was almost in agreement with the report of Dashe et al. [17] from Nigeria who reported 15.8% isolation rate of *E. coli* from liver and 13% from spleen suggesting that *E coli* localizes most commonly in these organs. This study shows the systemic infection of backyard chickens due to *E. coli*. The isolation rate was relatively higher in the spleen and liver, probably due to the reason that the role of the former as lymphoid organ for filtration of pathogens in chickens and the later due to retention of bacteria during the portal circulation and hepatic filtration system sequentially before any other peripheral organ.

Considering all the 191 chickens and 694 organ samples, the chicken and organ level isolation of *E. coli* was 32.5 and 11.5%, respectively. The chicken level isolation rate in the current study was nearly in-line with the report of Robert et al. [18] from Thailand, who reported 39% isolation rate of *E. coli* from cloacal and carcass swabs but higher than 18% report by Gokben and Adile [19] in Turkey. However, the current finding was lower than what has been documented by Abu saim et al [20], who reported isolation rate of 83.3% from poultry faeces and meat. As *E. coli* is a member of the normal microflora of the intestine of poultry and other animals, the isolation rate from faeces and carcass surface (due to contamination) could be higher [4]. Accordingly, the low prevalence in the present study might be due to consideration of organ samples which are free of any external contamination. Moreover, the variation among the studies might also be due to differences in environmental factors, feeding habits, presence or absence of concurrent infections, the standard of management and antibiotics usage.

The study indicated significantly higher (*P* < 0.05) *E. coli* isolation rate in adult (37.8%) than young (23.6%) chickens. This was consistent with the work of Rahman et al. [7] who also reported isolation rate of 36.7% from adult chickens in Bangladesh. Accordingly, the high prevalence of *E. coli* in adult chickens than young ones could be attributed to the fact that adult chickens have a much longer exposure time to infection. The isolation rate of 31.4 and 33.1% in male and female chickens, respectively in the present study was in agreement with the report of Zanella et al. [21]. Though there is sampling disproportion in the present study, the absence of significant difference (*P* > 0.05) in the isolation rate of *E. coli* between the two sexes indicate that both sexes are equally susceptible and there is same chance of exposure to risk of infection. On the contrary, high isolation rate was reported in layers than males [22].

There was significant association of *E. coli* isolation rate with clinically sick (42.7%) than apparently healthy chickens (22.1%). This is similar to isolation rate of 42% from samples of chickens with colisepticaemia [23]. This could be due to the fact that chickens with compromised immune system due to other diseases are usually more susceptible to various diseases including colibacillosis caused by *E. coli* or the bacteria itself might act as potentially important avian pathogen causing illness.

In this study, *E. coli* isolates showed varying level of antimicrobial susceptibility comparable to the previous findings of Guerra et al. [24]. *E. coli* isolates were completely (100%) susceptible to ciprofloxacin, norfloxacin and sulfamethoxazole-trimethoprim and majority of the isolates were also susceptible to gentamicin (93%), streptomycin (85%), nalxidic acid (83%), kanamycin (75%), and chloramphenicol (59%). These findings were in close agreement with the results of Shecho et al. [25] who reported 100 and 92.3% susceptibility of *E. coli* isolates to ciprofloxacin and sulfamethoxazole-trimethoprim, respectively in Ethiopia. Amare et al. [26] reported 100% susceptibility of *E. coli* to gentamicin and chloramphenicol from Ethiopia. A relatively higher susceptibility to gentamicin (87%) in Uganda [27], kanamycin (85.7%) in Bangladesh [20] and chloramphenicol (77%) in Bangladesh [28] were also reported. However, the current finding contradicts with the results of Zahraei and Farashi [29] and Zakeri and Kashefi [30] who recorded highly resistant *E. coli* isolates to nalidxic acid (100%), kanamycin (77%), streptomycin (67%) and chloramphenicol (67%). This may be due to the variation in the use of these antimicrobial drugs in various regions and the available parenteral preparation might not be prescribed for use in humans and animals including chickens or due to presence of different clones of *E. coli* in the study area.

Now a day’s antimicrobial resistance has become a worldwide concern [31]. This might be due to indiscriminate use of antimicrobials in human medicine, veterinary, and agriculture that promote the emergence and distribution of antimicrobial resistant microorganisms [32]. In the current study, *E. coli* isolates showed complete resistance to cloxacilin, amoxicillin and cefuroxime and moderate to high level of resistance (46.3–92.7%) to cefotaxime, cotrimoxazole and tetracycline. This resistance pattern was almost similar to the report of Nazir et al. [33] and Hossain et al. [28] from Bangladesh who reported 100% resistance to cloxacilin. In accord with the present result, *E. coli* isolated from various visceral organs, carcass and cloacal swab of chicken have also been reported to have 45% to tetracycline [34]. Robert et al. [18] from Thailand reported resistance of *E. coli* isolates from different chicken visceral organs to amoxicillin (73.3%).

In the present study MDR to two to four antimicrobial classes appeared to be the most common among MDR *E. coli*, which is in close agreement with Adenipekun et al. [35]. The high level of antimicrobial resistance observed within or between antimicrobial classes in various studies might be due to the widespread, indiscriminate, and lengthy use of similar drugs in the poultry farms [36]. In this study, the observed antimicrobial resistance of *E. coli* isolates is from chickens of backyard origin, which are less commonly treated with formally prescribed antimicrobials as compared to chickens under intensive management system. However, according to the information from some backyard chicken owner, it is a common practice to treat sick chickens using drugs such as oxytetracycline powder and other drugs which they didn’t know their name specifically that are obtained from open market or veterinary/ medical pharmacies. Such practice of using antimicrobials by untrained local people for treatment of chickens without proper diagnosis, selection of appropriate antimicrobial drugs, and strict adherence to proper dosage and frequency of administration, could result in development of antimicrobial resistance. It might also be due to the widespread use of antimicrobials in humans and other livestock species, or incorrect use of antimicrobials by the rural people and chicken may ingest the antimicrobial residuals from human and animal wastes or due to improper disposal of leftover antimicrobials by rural people after getting relief from their disease. In addition, plasmid mediated with a variety of genetic factors might also contribute to resistance in these antimicrobials [37], which could make it more possible for a susceptible bacterium to acquire resistance factors through conjugation or transformation [31].

In this study, as a limitation, sampling few exotic/hybrid chicken breeds made comparison with local chicken less sound. In addition, failure to undertake molecular tests to delineate those isolates with virulence and resistance genes were not assessed so that their role on pathogenesis could be justified. The risk factor for drug resistance was also not properly addressed, due to inability to get enough information from people who brought chickens to market.

## Conclusions

The present study evidenced the presence of a considerable *E. coli* isolates in various organs of clinically sick than apparently healthy chickens. Age and health status of chickens were the risk factors for *E. coli* infection. Substantial proportion of *E. coli* isolates were found resistant to different classes of antimicrobial drugs, which could have an important public health consequence if they get access to humans. Therefore, control of irrational use of antimicrobial in humans and farm animals including limiting the availability of antimicrobials in the illegal market needs to be addressed. Moreover, establishment of guidelines for prudent use of antimicrobials in farm animals with effective enforcement is required in Ethiopia. Measures such as improving backyard chicken farming practices and educating the rural community to build up a knowledge base on the antimicrobial resistance and its impact on veterinary and public health is suggested.

## Additional files

Additional file 1: Data for Chicken, E.coli 2017. (XLSX 34 kb)

## Abbreviations

aOR: adjusted Odds Ratio
CI: Confidence Interval
EMB: Eosin Methylene Blue agar
IMViC: Indole production, Methyl-red, Voges- Proskauer, Citrate utilization tests
MDR: Multi Drug Resistance
TSI: Triple Sugar Iron
